# Obesity is Associated With Delayed Graft Function in Kidney Transplant Recipients: A Paired Kidney Analysis

**DOI:** 10.3389/ti.2023.11107

**Published:** 2023-05-30

**Authors:** Bree Shi, Tracey Ying, Josephine Xu, Kate Wyburn, Jerome Laurence, Steven J Chadban

**Affiliations:** ^1^ Charles Perkins Centre, The University of Sydney, Camperdown, NSW, Australia; ^2^ Australia and New Zealand Dialysis and Transplant (ANZDATA) Registry, South Australia Health and Medicine Research Institute, Adelaide, SA, Australia; ^3^ Renal Medicine, Royal Prince Alfred Hospital, Camperdown, NSW, Australia; ^4^ Department of Transplant Surgery, Royal Prince Alfred Hospital, Camperdown, NSW, Australia; ^5^ Institute of Academic Surgery, Royal Prince Alfred Hospital, Sydney, NSW, Australia; ^6^ School of Public Health, Faculty of Medicine and Health, The University of Sydney, Sydney, NSW, Australia

**Keywords:** kidney transplantation, patient survival, graft survival, obesity, DGF

## Abstract

Obesity is increasingly prevalent among candidates for kidney transplantation. Existing studies have shown conflicting post-transplant outcomes for obese patients which may relate to confounding bias from donor-related characteristics that were unaccounted for. We used ANZDATA Registry data to compare graft and patient survival between obese (BMI >27.5 kg/m^2^ Asians; >30 kg/m^2^ non-Asians) and non-obese kidney transplant recipients, while controlling for donor characteristics by comparing recipients of paired kidneys. We selected transplant pairs (2000–2020) where a deceased donor supplied one kidney to an obese candidate and the other to a non-obese candidate. We compared the incidence of delayed graft function (DGF), graft failure and death by multivariable models. We identified 1,522 pairs. Obesity was associated with an increased risk of DGF (aRR = 1.26, 95% CI 1.11–1.44, *p* < 0.001). Obese recipients were more likely to experience death-censored graft failure (aHR = 1.25, 95% CI 1.05–1.49, *p* = 0.012), and more likely to die with function (aHR = 1.32, 95% CI 1.15–1.56, *p* = 0.001), versus non-obese recipients. Long-term patient survival was significantly worse in obese patients with 10- and 15-year survival of 71% and 56% compared to 77% and 63% in non-obese patients. Addressing obesity is an unmet clinical need in kidney transplantation.

## Graphical Abstract

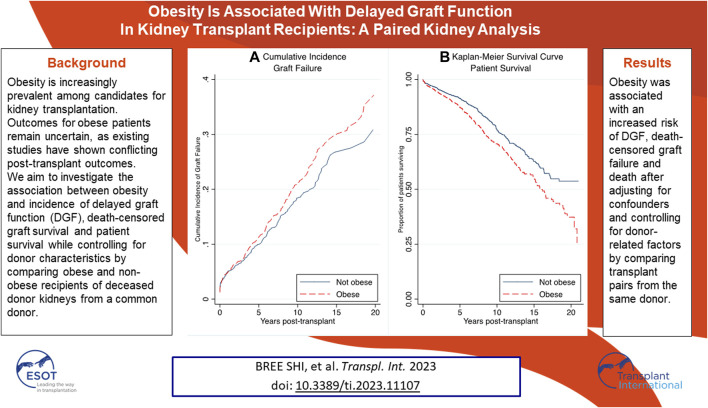

## Introduction

Over the past four decades, the worldwide prevalence of obesity has tripled. In addition to the associations between obesity and hypertension, type 2 diabetes and coronary artery disease, obesity is clearly associated with premature mortality [1]. Obesity has therefore had a significant impact on community health as well as posing a major economic challenge to global healthcare systems. Obesity is increasingly prevalent in the end-stage kidney disease (ESKD) and kidney transplant populations [2,3]. In the US, the proportion of ESKD patients that were obese between 2008 and 2016 was nearly 40% [4].

Whilst kidney transplant recipients with high body mass index (BMI) are more like to develop post-transplant diabetes, congestive heart failure, atrial fibrillation, and cardiovascular death [5–7], transplantation offers a survival benefit for obese recipients compared to remaining on dialysis [8,9]. However, kidney transplant recipients with high BMI are at an increased risk of post-transplant complications, including prolonged wound healing, dehiscence, hernias, surgical site infections, deep vein thrombosis, and reintubation. These issues contribute to a longer hospital stay and higher hospital costs for transplantation in the obese [10–12].

The long-term graft and patient outcomes of obese recipients compared to non-obese recipients have remained controversial. When compared to non-obese recipients, some reports described an increased risk of graft failure and mortality for obese recipients whilst others have found no significant differences [12–15]. These disparate outcomes, may relate to the confounding bias of non-randomly distributed donor-related characteristics which were not accounted for [16–18]. Therefore, we sought to investigate the association between obesity and incidence of delayed graft function (DGF), graft survival and patient survival while controlling for donor characteristics by comparing obese and non-obese recipients of kidneys from a common donor, a matched-pair analysis. We hypothesized that obesity would increase the risk of DGF and lead to inferior graft and patient survival.

## Materials and Methods

We extracted data from the Australia and New Zealand Dialysis and Transplant Registry (ANZDATA). The ANZDATA Registry is a clinical quality registry that collects comprehensive data from all patients with ESKD in Australia and New Zealand. Details of the structure and method of ANZDATA Registry data collection can be found on the Registry website (https://www.anzdata.org.au/anzdata/). We included all deceased donor kidney-only transplant pairs between 1 January 2000 and 31 December 2020, where a deceased donor supplied one kidney to an obese recipient and the other to a non-obese recipient. We excluded recipients under the age of 18, recipients of a deceased donor kidney retrieved outside Australia or New Zealand, and recipients of a second or subsequent transplant. We used the World Health Organization (WHO) classification of obesity as BMI greater than 30 kg/m^2^ for non-Asians, and greater than 27.5 kg/m^2^ for Asians due to differences in body habitus compared to the Western population [19–21]. Follow-up was until loss to follow-up, or 31 December 2020. The primary outcome was DGF which was defined as receipt of hemodialysis within 72 h after transplant prior to 2017, and receipt of hemodialysis within 7 days of transplantation after 2017 [22]. This modification to the definition of delayed graft function was due to a policy change made by ANZDATA in 2017. The secondary outcomes were death and death-censored graft failure.

We compared baseline characteristics of paired recipients using paired t-test or Wilcoxon’s signed rank test for continuous variables and McNemar’s test for dichotomous variables. We estimated the cumulative incidence of graft failure using Aalen-Johansen estimator to account for death as a competing event. We used Gray’s test to compare the cumulative incidence of graft failure in the presence of the competing risk of death. We used Kaplan-Meier curves to compare unadjusted patient survival. We used a logrank test to compare the probability of patient survival at different time points. We estimated the rate ratio of DGF for obese patients compared with non-obese patients, using conditional Poisson regression, adjusting for potential confounders [23–25]. As a sensitivity analysis, we repeated this analysis excluding patients who experienced graft failure within 90 days of transplantation. Time to graft failure and time to death were analyzed using Cox regression stratified by donor [24,25].

A dose-response analysis was performed to examine the association between the degree of obesity (i.e., class I, class II and class III) and clinical outcomes. Obesity was categorized as class I, class II and class III according to WHO guidelines (Table 3). We estimated the rate ratio of DGF using conditional Poisson regression and the hazard ratio of graft failure and death using Cox regression, adjusting for potential confounders.

The potential confounders considered were age at transplantation, sex, ethnicity, cause of kidney disease, duration of dialysis, dialysis modality prior to transplant, human leukocyte antigen (HLA) mismatch, ischemia time, maximum panel reactive antibodies, donor kidney side, pre-existing comorbidities including diabetes, chronic lung disease, cardiovascular disease (any of coronary artery, cerebrovascular or peripheral vascular), and non-skin cancer, acute rejection within 6 months of transplantation (for graft failure and death only), DGF (as a categorical variable, for graft failure and death only), and graft failure (as a time-varying covariate, for death only). We used stepwise selection methods where variables with a significance level of 0.20 were considered and included in the base multivariable model. We used backward selection method to remove variables that were not significant at the 0.05 level [26]. We used complete case analysis because the number of missing values was less than 5%. All analyses were performed using Stata Statistical Software: Release 14.2 (StataCorp., College Station, TX) This study was approved by the Ethics Review Committee of the Sydney Local Health District, Royal Prince Alfred Hospital Zone.

## Results

### Study Cohort

**FIGURE 1:**
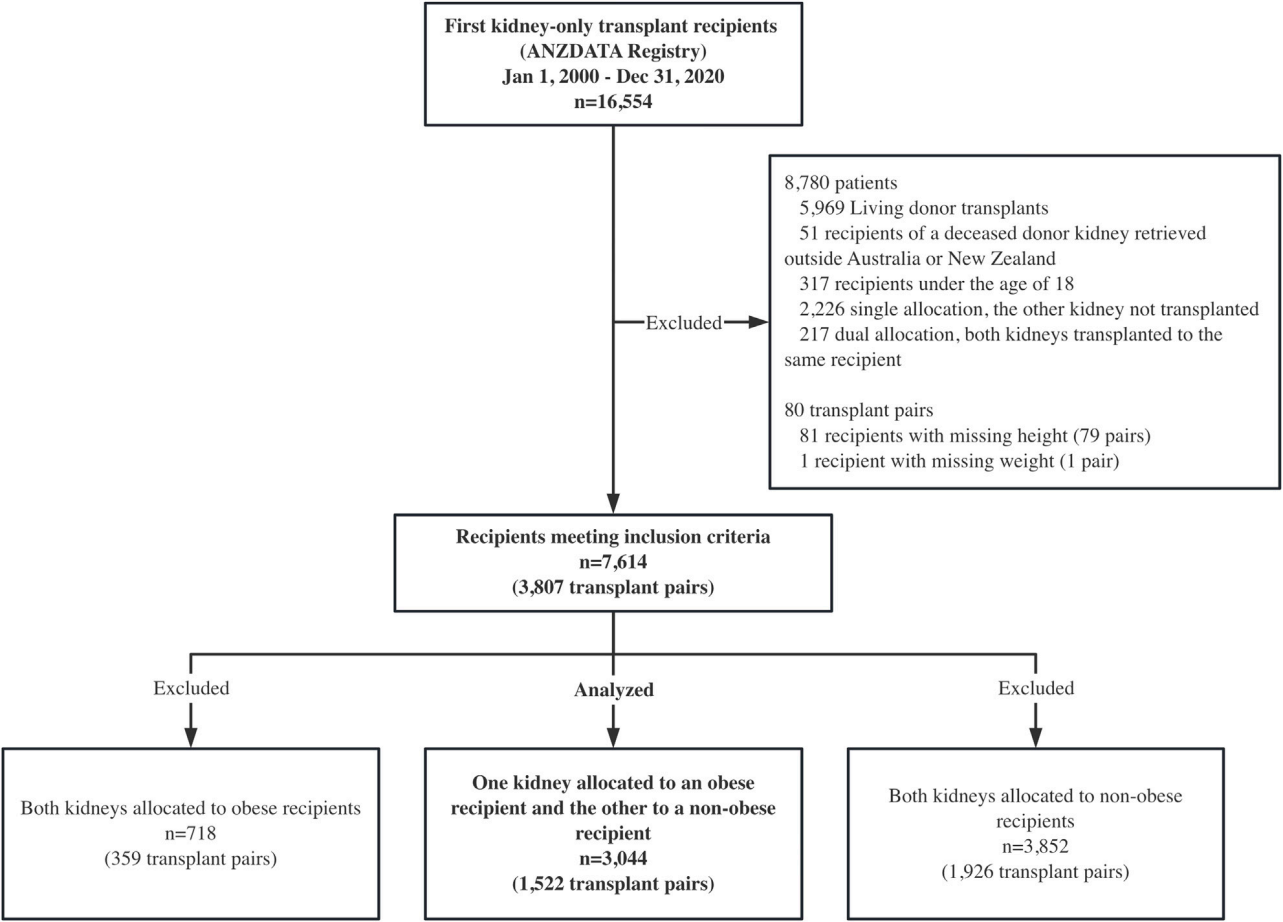
Study design: a paired kidney analysis where one kidney was allocated to an obese recipient and the other to a non-obese recipient. ANZDATA, the Australia and New Zealand Dialysis and Transplant registry.

Between 1 January 2000, and 31 December 2020, 16,554 patients received their first kidney transplant in Australia and New Zealand. After inclusion and exclusion criteria were applied, 1,522 pairs were identified where a deceased donor supplied one kidney to an obese recipient and the other to a non-obese recipient (Figure 1). Follow-up time was 19,768 person-years in total, with a median follow-up time of 5.3 years (interquartile range 2.5–9.5 years). Nine of the obese recipients and seven of the non-obese recipients were lost to follow-up.

Donor and recipient baseline characteristics are summarized in Tables 1, 2. Baseline characteristics indicate that obese and non-obese recipients were comparable in terms of sex, time on dialysis, ischemia time, HLA mismatch and maximum panel reactive antibody percentage. The obese group included a higher proportion of recipients aged 50–65 (48% vs. 44%), *p* < 0.001), fewer people of Asian ancestry (12% vs. 15%, *p* < 0.001), more Indigenous people (17% vs. 11%, *p* < 0.001), more people with pre-existing diabetes (33% vs. 21%, *p* < 0.001) and comorbid cardiovascular disease (33% vs. 27%, *p* = 0.001) and more right-sided kidneys (55% vs. 45%, *p* < 0.001).

**TABLE 1:** Donor characteristics.

Factor	N = 1,522 n (%)
Age	
<18	79 (5)
18–34	254 (17)
35–49	426 (28)
50–65	556 (37)
65+	207 (14)
Male	870 (57)
Body mass index (BMI)	
Underweight	47 (3)
Normal	556 (37)
Overweight	534 (35)
Obese	383 (25)
Terminal serum creatinine concentration, μmol/L	96.4 ± 83.2
Diabetes	96 (6)
Hypertension	388 (25)
Neurological determination of death (NDD)	1,167 (77)
Cause of death	
Intracranial hemorrhage	640 (44)
Traumatic brain injury	285 (19)
Cerebral infarct	94 (6)
Cerebral hypoxia/ischemia	380 (26)
Other neurological condition	12 (1)
Non-neurological condition	59 (4)

**TABLE 2:** Recipient and transplantation characteristics for obese and non-obese recipients.

Factor	Obese	Not obese	*p*-value
	N = 1,522	N = 1,522	
Age at transplant	n (%)	n (%)	<0.001
18–34	113 (7)	193 (13)	
35–49	430 (28)	414 (27)	
50–65	728 (48)	667 (44)	
65+	251 (16)	248 (16)	
Male	985 (65)	991 (65)	0.82
Ethnicity			<0.001
Caucasian	1,001 (66)	1,006 (66)	
Indigenous	257 (17)	162 (11)	
Asian	185 (12)	232 (15)	
Other	79 (5)	122 (8)	
Primary renal disease			<0.001
GN	575 (38)	628 (41)	
Renovascular	123 (8)	112 (7)	
Diabetes	351 (23)	231 (15)	
Other	473 (31)	551 (36)	
Time since first RRT			0.14
0–1 year	173 (11)	209 (14)	
1–3 years	594 (39)	575 (38)	
Over 3 years	755 (50)	738 (48)	
Dialysis modality prior to transplant			0.008
Pre-emptive transplant	11 (1)	17 (1)	
HD	1,106 (73)	1,030 (68)	
PD	405 (27)	475 (31)	
Ischemia time [mean (sd)]	12.1 (4.9)	12.0 (5.0)	0.52
HLA mismatches			0.58
0	46 (3)	38 (2)	
1–2	408 (27)	427 (28)	
3–4	483 (32)	460 (30)	
5–6	580 (38)	596 (39)	
Maximum panel reactive antibodies			0.50
0	918 (60)	935 (61)	
1–50	491 (32)	465 (31)	
>50	110 (7)	121 (8)	
Pre-existing comorbidities			
Chronic lung disease	130 (9)	124 (8)	0.69
Cardiovascular disease	501 (33)	418 (27)	0.001
Diabetes	504 (33)	316 (21)	<0.001
Right kidney	832 (55)	690 (45)	<0.001

GN, Glomerulonephritis; HD, hemodialysis; PD, peritoneal dialysis; HLA, Human Leukocyte Antigen; RRT, renal replacement therapy.

### Outcomes

#### Delayed Graft Function

A greater proportion of obese recipients experienced DGF compared to non-obese recipients (39% vs. 30%, *p* < 0.001). Conditional Poisson regression demonstrated an increased risk of DGF for obese recipients versus their non-obese pair (aRR = 1.27, 95% CI 1.12–1.44, *p* < 0.001), after adjusting for dialysis modality prior to transplant, ischemia time and pre-existing cardiovascular disease and accounting for donor-related factors (Supplementary Table S1).

Sensitivity analysis, excluding those patients who experienced graft failure within 90 days of transplantation, showed a similar effect of obesity on DGF to the primary analysis (aRR = 1.29, 95% CI 1.12–1.48, *p* < 0.001).

#### Graft Failure

**FIGURE 2:**
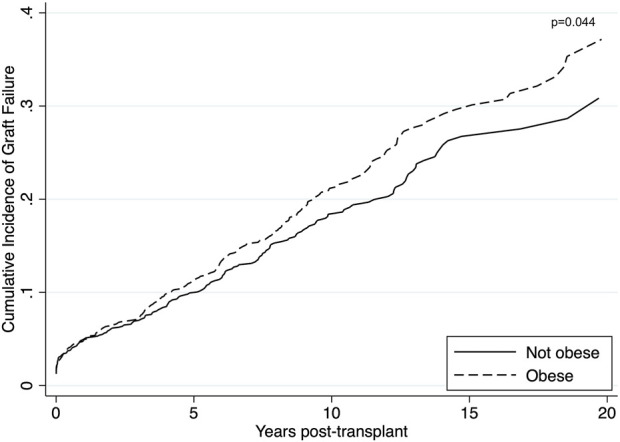
Unadjusted cumulative incidence of graft failure using Aalen-Johansen estimator to account for death as a competing event among obese (BMI >27.5 kg/m^2^ for Asians; >30 kg/m^2^ for non-Asians) and non-obese recipients. Obese recipients had a higher incidence of graft failure compared to non-obese recipients (*p* = 0.044).

Unadjusted graft failure was more common amongst obese recipients (Figure 2). Cumulative incidence of graft failure at 5 years was not affected by obesity status (11% obese vs. 10% non-obese), however, obese recipients were found to have a higher incidence of long-term graft failure with 10- and 15-year cumulative incidence of 21% and 30% compared to 18% and 27% in non-obese patients. The Gray’s test confirmed a significant difference on the overall incidence of graft failure between obese and non-obese recipients (*p* = 0.044). On multivariable analysis, obesity was confirmed as an independent risk factor for death-censored graft failure. Obesity was associated with a higher risk of death-censored graft failure after adjusting for DGF, donor kidney side, age, ethnicity and HLA mismatch (aHR = 1.25, 95% CI 1.05–1.49, *p* = 0.012) (Supplementary Table S2). Recipients who experienced delayed graft function were more likely to experience death-censored graft failure (aHR = 1.84, 95% CI 1.39–2.44, *p* < 0.001).

#### Patient Survival

**FIGURE 3:**
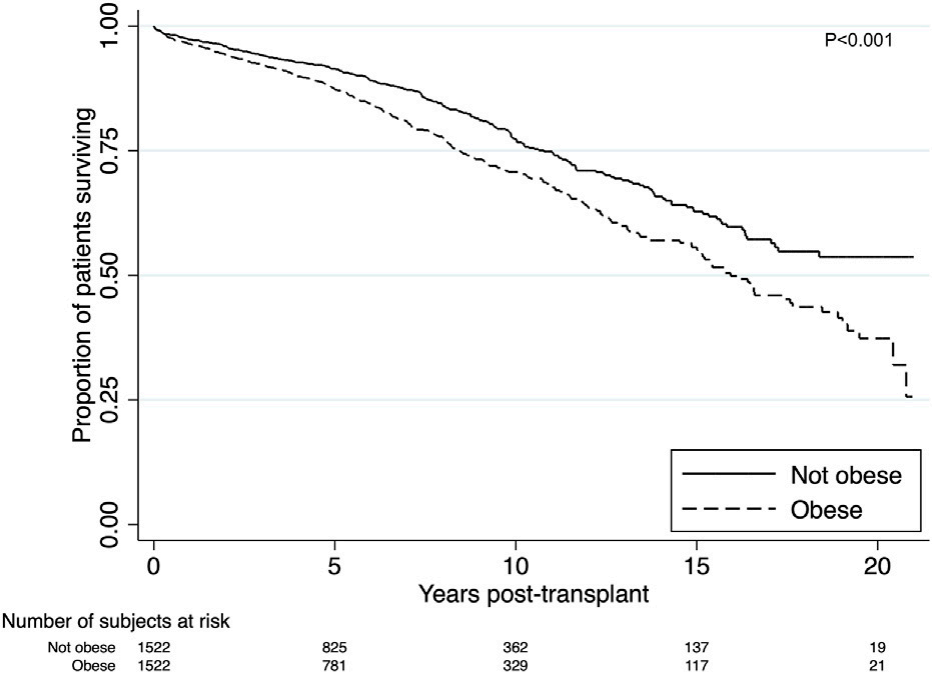
Kaplan-Meier estimates of patient survival after renal transplant among obese (BMI >27.5 kg/m^2^ for Asians; >30 kg/m^2^ for non-Asians) and non-obese recipients. Unadjusted patient survival was superior for non-obese recipients (*p* < 0.001).

There were 342 (22%) deaths in the obese group compared to 260 (17%) (*p* < 0.001). Death from cardiovascular disease was the most prominent cause of death amongst the obese recipients, with 105 cardiovascular deaths (31%) compared to 65 (25%) among the non-obese recipients. Obesity was strongly associated with inferior survival in both the short and long-term (*p* < 0.001) (Figure 3). Short and long-term patient survival was significantly worse in obese recipients with 5-, 10- and 15-year survival of 87%, 71% and 56% compared to 91%, 77% and 63% in non-obese patients (*p* = 0.017, *p* < 0.001, *p* < 0.001). In the multivariable model, obesity was found to be strongly associated with worse patient survival. Obese recipients had an increased risk of death compared to non-obese recipients (aHR = 1.32, 95% CI 1.15–1.56, *p* = 0.001) (Supplementary Table S3). Significant determinants of death that were included in the final model were graft failure, older age, Indigenous ethnicity, diabetes as primary renal disease, length of time on dialysis and pre-existing cardiovascular disease. Graft failure was adjusted as a time-varying covariate in the model. Recipients with graft failure had a much higher risk of death (aHR = 2.84, 95% CI 2.00–4.03, *p* < 0.001).

#### Degree of Obesity and Clinical Outcomes

We performed a dose-response analysis to examine the association between the degree of obesity and clinical outcomes. The 1,522 obese recipients were classified as 1,173 (77%) class I; 304 (20%) class II and 45 (3%) class III (Table 3). We combined obesity classes II and III due to insufficient patient numbers in obesity class III.

**TABLE 3:** Degree of obesity was categorized into obese class I, obese class II, and obese class III according to World Health Organization guidelines.

Classification	BMI, kg/m^2^, non-Asians	BMI, kg/m^2^, Asians	n (%)
Obese class I	30–34.9	27.5–32.4	1,173 (77)
Obese class II	35–39.9	32.5–37.4	304 (20)
Obese class III	40+	37.5+	45 (3)

**FIGURE 4:**
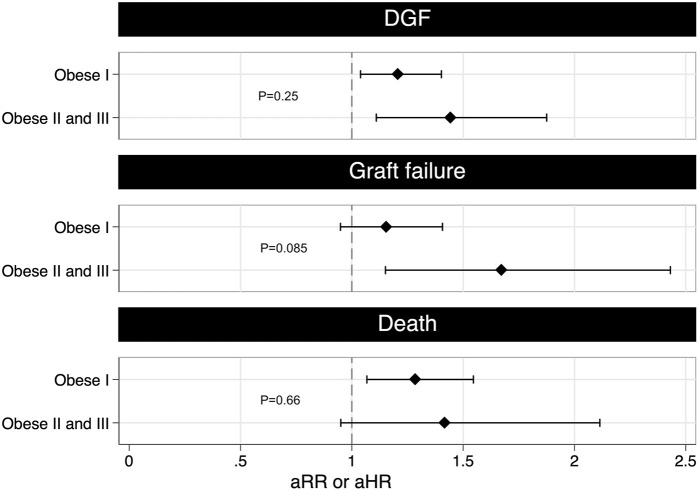
The figure shows the multivariable adjusted risk ratio for DGF and adjusted hazard ratio for graft failure and death grouped by class I obese and class II and III obese. A higher risk of DGF and graft failure was found for greater vs. lesser degrees of obesity, suggesting a possible dose-response relationship (DGF: aRR 1.20, 95% CI 0.88–1.62, *p* = 0.25; graft failure: aHR 1.45, 95% CI 0.95–2.21, *p* = 0.085). The adjusted rate ratio for DGF was estimated using conditional Poisson regression after adjusting for dialysis modality prior to transplant, ischemia time and pre-existing cardiovascular disease. The adjusted hazard ratio for graft failure was estimated using stratified Cox regression after adjusting for DGF, age at transplantation, ethnicity, HLA mismatches and donor kidney side. The adjusted hazard ratio for death was estimated using stratified Cox regression after adjusting for graft failure, age at transplantation, time since first renal replacement therapy, HLA mismatches, pre-existing cardiovascular disease and pre-existing diabetes. aHR, adjusted hazard ratio; aRR, adjusted rate ratio; DGF, delayed graft function; HLA, Human Leukocyte Antigen.

When comparing with non-obese recipients, class II/III obese recipients had a 1.44 higher rate of DGF whilst class I obese recipients had a 1.20 higher rate. This trend was not statistically significant when comparing class I obese recipients to class II/III obese recipients (Figure 4. aRR 1.20, 95% CI 0.88–1.62, *p* = 0.25). A similar non-significant trend was found for death-censored graft failure and death. Class II/III obese recipients had a 1.67 higher rate of death-censored graft failure compared to a 1.16 higher rate for class I obese recipients (aHR 1.45, 95% CI 0.95–2.21, *p* = 0.085). Class II/III obese recipients had a 1.42 higher rate of death compared to a 1.26 higher rate for class I obese recipients (aHR 1.10, 95% CI 0.71–1.71, *p* = 0.66).

## Discussion

In this paired analysis, we controlled for unmeasured donor-related characteristics by comparing outcomes of kidneys from the same donor and demonstrated that obese recipients were more likely to experience DGF, death-censored graft failure and death after deceased donor kidney transplantation when comparing with non-obese recipients.

Studies examining the impact of obesity on kidney transplant outcomes have shown conflicting results, but may be confounded by unmeasured donor-related characteristics. These may include donor kidney function and proteinuria, pre-renal insults to the donor kidney during terminal illness, use of inotropic medications and nephrotoxin exposure, many of which are not adequately captured nor accounted for in existing studies, The majority of published studies have reported an increased risk of delayed graft function for obese recipients [12,13,15]. However, the impact of DGF on long-term transplant outcomes including graft and patient survival remains contentious. Our results are consistent with two systematic review and meta-analyses which showed an increased risk of graft failure for obese recipients compared to non-obese recipients [14,15]. In terms of overall mortality, two meta-analyses reported an increased risk of death for obese recipients, in line with our results [12,14]. One systematic review and meta-analysis reported no association between obesity and overall mortality [27], however, this analysis included only six studies that reported hard transplant outcomes. Another systematic review and meta-analysis reported that there was an increased risk of graft failure and death only for studies that included obese patients who were transplanted before 2000, but no association for those transplanted after 2000 [13]. This contradicts our study result which included patients transplanted after 2000 only.

We found a trend towards increasing risks of DGF, graft failure and death with increasing degrees of obesity, however, this increase was not statistically significant. The number of recipients with class II/III obesity in our study was small and likely inadequately powered to provide certainty. A recent US registry study reported 27% lower odds of DGF (*p* < 0.001) for recipients with BMI >30–35 versus BMI >35 kg/m^2^, though no difference in graft or patient survival at a median follow-up of 3.9 years [28].

Our study provides detailed insights from a large, bi-national kidney transplant registry over a 20-year period. We examined a different BMI cut-off for the Asian population that has significant structural variations compared to the Western population. Donor-related factors, which could potentially impact outcomes such as DGF, were carefully accounted and unmeasured confounders were evenly matched by the use of a matched-pair analysis. As randomized controlled trials to compare outcomes for obese versus non-obese recipients are not feasible, we believe the paired analysis we have performed provides the most rigorous assessment of the impact of obesity on hard outcomes following kidney transplantation.

Obesity has more than doubled worldwide in the past 20 years. Although our study has demonstrated that obesity was strongly associated with an increased risk of DGF and inferior long-term outcomes, previous work has clearly indicated that transplantation yields superior outcomes compared to remaining on dialysis for the majority of obese candidates for transplantation [8,9,29]. Our findings should be used to inform patients and providers of the increased risks associated with transplantation for obese recipients. Rather than avoiding transplantation for the obese, these data should encourage the pursuit of strategies to improve outcomes, such as weight-loss management prior to transplantation and improvements in peri-operative management to reduce the incidence of DGF and other complications associated with obesity. This poses two key questions: (1) can transplant management be optimized for obese recipients; and (2) can weight loss before or post-transplant improve transplant outcomes for obese candidates. Some studies have reported an “obesity paradox” where a decrease in BMI for dialysis patients was associated with worse graft and patient survival [30–33]. However, in these studies there was no clear indication of whether the weight loss was intentional, or unintentional due to disease progression or comorbidities. The reason behind the paradox remains unknown. Hypotheses include that obese patients may be less prone to protein energy wasting [34], have a better appetite and well-preserved energy stores, have better hemodynamic tolerance, stem cell mobilization, hemodynamic tolerance, and more efficient disposal of lipophilic uremic toxins [35,36]. A healthy lifestyle that is beneficial to the general public has been shown to improve mortality in chronic kidney disease (CKD) patients [37]. Intentional weight loss in the pre-transplant population may reduce the risk of wound infection, DGF, death-censored failure and reduce the length of hospitalization and alleviates the financial burden on transplant programs [38]. Weight-management programs for CKD patients that include a renal-specific diet, regular exercise combined with anti-obesity medication have been reported to be effective in weight reduction, with improved functional ability, graft function and significantly longer adverse event-free period for the combined outcome of all-cause mortality, myocardial infarction, stroke, and hospitalization for congestive heart failure [39–41]. Another possible intervention is bariatric surgery. A recent study reported a lowering of 7 kg/m^2^ in BMI in the long-term and a median of 2.4 years longer life expectancy in the bariatric surgery cohort compared to usual obesity care [42]. However, there is very limited data on the outcomes of bariatric surgery on dialysis and kidney transplant patients. In a retrospective cohort study, researchers demonstrated lower all-cause mortality at 5 years for obese ESKD patients who had undergone bariatric surgery [43]. In another retrospective study, bariatric surgery before or after kidney transplantation was reported to be associated with reduced risk of graft failure and mortality compared to control with no bariatric surgery [44]. More data are required to determine if bariatric surgery does improve long-term outcomes from kidney transplantation.

Several limitations should be noted in considering our analysis. First, it is a retrospective registry study that depends on the quality of data captured. Second, the analysis used BMI as the only indicator for categorizing obesity, which does not differentiate between fat and muscle mass, nor between visceral and subcutaneous fat. Other methods such as waist circumference, waist-to-hip ratio, *in vivo* neutron activation analysis (IVNAA), densitometry, deuterium oxide dilution, and dual energy X-ray absorptiometry (DXA) are also available and may enhance specificity. However, such measures are not routinely used in candidate assessment and are not reported to ANZDATA. Third, there may be other potential confounders that are unaccounted for, such as social status, genetic factors, immunosuppression and drug dosing. Fourth, even though significant confounders were adjusted for in the model, residual confounding is still possible. Five, indication of whether dialysis is required after transplantation may vary between centers resulting in potential center effect for DGF which was not accounted for. Six, there may be a loss of statistical power due to pairing. However, we believe that it is important to utilize a matched pair analysis to minimize bias due to donor-related characteristics, such as donor kidney function, hemodynamic instability during organ procurement, use of vasoactive medications and exposure to nephrotoxins, all of which are captured crudely or not at all in registry data. Finally, the study cohort was predominantly Caucasian. The remaining non-Caucasian patient group was heterogeneous, with 40% and 23% of the Indigenous group being Australian Aboriginal and New Zealand Mauri, and 25% and 23% of the Asian group being Indian and Chinese, respectively. Therefore, the comparison between Caucasian and non-Caucasian patients in our study is different from the same comparison in the US where around 70% of non-Caucasian patients were Black/African American [45].

In conclusion, our study demonstrates a relationship between obesity and post-transplant outcomes after carefully controlling for donor-related factors in a paired kidney analysis. Addressing obesity is an unmet clinical need in kidney transplantation. Transplantation is recommended for many obese candidates as it is acknowledged to yield superior outcomes to dialysis. However, design and evaluation of strategies to: (1) optimize transplant management for obese recipients; and (2) reduce the prevalence of obesity among transplant candidates are required.

## Data Availability

The data analyzed in this study is subject to the following licenses/restrictions: The data are not available due to privacy and ethical considerations. Requests to access these datasets should be directed to https://anzdata.org.au/.

## Abbreviations

aHR, adjusted hazard ratio; ANZDATA, the Australia and New Zealand Dialysis and Transplant registry; aRR, adjusted rate ratio; BMI, body mass index; CI, confidence interval; CKD, chronic kidney disease; DGF, delayed graft function; ESKD, end-stage kidney disease; GN, Glomerulonephritis; HD, hemodialysis; HLA, human leukocyte antigen; PD, peritoneal dialysis; RRT, renal replacement therapy; WHO, World Health Organization.
