# Supplementary material for: Obesity is Associated With Delayed Graft Function in Kidney Transplant Recipients: A Paired Kidney Analysis
Source: Transpl Int. 2023 May 30;36:11107. doi: 10.3389/ti.2023.11107 (PMC10261700; doi:10.3389/ti.2023.11107)
Supplement: Supplementary file 1 [file DataSheet1.docx]

Supplementary Table of Contents:

Table S1: Risk factors for delayed graft function. The multivariable conditional Poisson regression model for delayed graft function risk factors.

Table S2: Risk factors for graft failure. The multivariable Cox regression model for graft failure risk factors, stratified by donor.

Table S3: Risk factors for recipient death. The multivariable Cox regression model for recipient death risk factors, stratified by donor.

Table S1: Risk factors for delayed graft function. The multivariable conditional Poisson regression model for delayed graft function risk factors.

| Factor | Rate Ratio | 95% CI | P-value |
| --- | --- | --- | --- |
| Obese | 1.27 | (1.12-1.44) | <0.001 |
| Dialysis modality prior to transplant |  |  | <0.001 |
| HD | Reference |  |  |
| PD | 0.51 | (0.41-0.64) |  |
| Ischaemia time | 1.04 | (1.02, 1.07) | 0.001 |
| Pre-existing Cardiovascular disease | 1.23 | (1.01-1.51) | 0.042 |

Table S2: Risk factors for graft failure. The multivariable Cox regression model for graft failure risk factors, stratified by donor.

| Factor | Hazard ratio | 95% CI | P-value |
| --- | --- | --- | --- |
| Obese | 1.25 | (1.05-1.49) | 0.012 |
| Delayed graft function | 1.84 | (1.39-2.44) | <0.001 |
| Right kidney | 1.31 | (1.11-1.55) | 0.002 |
| Age at transplant |  |  |  |
| 18-34 | Reference |  | 0.002 |
| 35-49 | 0.53 | (0.36-0.77) |  |
| 50-65 | 0.48 | (0.32-0.70) |  |
| 65+ | 0.58 | (0.35-0.97) |  |
| Ethnicity |  |  |  |
| Caucasian | Reference |  | 0.003 |
| Indigenous | 1.21 | (0.83-1.77) |  |
| Asian | 0.56 | (0.37-0.86) |  |
| Other | 0.44 | (0.23-0.86) |  |
| HLA mismatches |  |  |  |
| 0 | Reference |  | <0.001 |
| 1-2 | 1.67 | (0.69-4.05) |  |
| 3-4 | 1.19 | (0.46-3.10) |  |
| 5-6 | 2.43 | (0.93-6.32) |  |

Table S3: Risk factors for recipient death. The multivariable Cox regression model for recipient death risk factors, stratified by donor.

| Factor | Hazard ratio | 95% CI | P-value |
| --- | --- | --- | --- |
| Obese | 1.32 | (1.15-1.56) | 0.001 |
| Graft failure | 2.84 | (2.00-4.03) | <0.001 |
| Age at transplant |  |  |  |
| 18-34 | Reference |  | <0.001 |
| 35-49 | 3.03 | (1.76-5.23) |  |
| 50-65 | 5.55 | (3.07-10.04) |  |
| 65+ | 8.68 | (4.50-16.73) |  |
| Ethnicity |  |  |  |
| Caucasian | Reference |  | 0.011 |
| Indigenous | 1.78 | (1.13-2.83) |  |
| Asian | 0.66 | (0.44-1.00) |  |
| Other | 0.99 | (0.57-1.70) |  |
| Primary renal disease |  |  |  |
| Glomerulonephritis | Reference |  | <0.001 |
| Renovascular | 1.16 | (0.76-1.78) |  |
| Diabetes | 2.19 | (1.53-3.14) |  |
| Other | 1.24 | (0.95-1.63) |  |
| Time since first RRT |  |  |  |
| 0-1yr | Reference |  | 0.007 |
| 1-3yr | 1.41 | (0.95-2.09) |  |
| 3yr+ | 1.84 | (1.25-2.70) |  |
| Pre-existing cardiovascular disease | 2.02 | (1.51-2.71) | <0.001 |
